# A Novel Deletion Mutation in ASPM Gene in an Iranian Family with Autosomal Recessive Primary Microcephaly

**Published:** 2013

**Authors:** Elinaz AKBARIAZAR, Mohammad EBRAHIMPOUR, Saeedeh AKBARI, Sanaz ARZHANGHI, Seydeh Sedigheh ABEDINI, Hossein NAJMABADI, Kimia KAHRIZI

**Affiliations:** 1 Msc of human genetics, University of Social Welfare & Rehabilitation Sciences, Tehran, Iran; 2 Bsc in Nursing, Genetics Research Center, University of Social Welfare & Rehabilitation Sciences, Tehran, Iran; 3 Professor of Molecular Biology, University of Social Welfare & Rehabilitation Sciences, Tehran, Iran; 4 Professor of Medical Genetics, Genetics Research Center, University of Social Welfare & Rehabilitation Sciences, Tehran, Iran

**Keywords:** Autosomal Recessive Primary Microcephaly, ASPM, MCPH5, Homozygosity Mapping

## Abstract

**Objective:**

Autosomal recessive primary microcephaly (MCPH) is a neurodevelopmental and genetically heterogeneous disorder with decreased head circumference due to the abnormality in fetal brain growth. To date, nine loci and nine genes responsible for the situation have been identified. Mutations in the ASPM gene (MCPH5) is the most common cause of MCPH. The ASPM gene with 28 exons is essential for normal mitotic spindle function in embryonic neuroblasts.

**Materials & Methods:**

We have ascertained twenty-two consanguineous families with intellectual disability and different ethnic backgrounds from Iran. Ten out of twenty-two families showed primary microcephaly in clinical examination. We investigated MCPH5 locus using homozygosity mapping by microsatellite marker.

**Result:**

Sequence analysis of exon 8 revealed a deletion of nucleotide (T) in donor site of splicing site of ASPM in one family. The remaining nine families were not linked to any of the known loci .More investigation will be needed to detect the causative defect in these families

**Conlusion:**

We detected a novel mutation in the donor splicing site of exon 8 of the ASPM gene. This deletion mutation can alter the ASPM transcript leading to functional impairment of the gene product.

## Introduction

Autosomal recessive primary microcephaly (MCPH; MIM 251200) is clinically characterized by an occipitofrontal head circumference (OFC) of at least three standard deviations (SDs) below the expected mean for age and sex(1-3). The disease is etiologically heterogeneous and environmental and genetic causes have both been identified as responsible causes of it. Environmental causes of MCPH are congenital infections, maternal alcohol consumption, and drug consumption during pregnancy (4,5). The major causes of microcephaly are genetic mechanisms including cytogenetic abnormalities and single gene disorders(5).

Microcephaly is divided into primary (present at birth) and secondary microcephaly (developing postnatal)(5). The birth prevalence of primary microcephaly varies from 1.3 to 150 per 100000 live births depending on the population (6). The birth prevalence of primary nonsyndromal microcephaly is 1:30000 to 1:250000 live births (6, 7). Until 2010, MCPH was reported in about 100 families worldwide; but, Darvish and colleagues investigated seven MCPH loci in patients with primary microcephaly from 112 consanguineous Iranian families (2).To date, nine MCPH loci and nine genes have been identified to be responsible for the situation. These genes include Microcephalin at MCPH1 (2, 8), WDR62 at MCPH2 (9, 10), CDK5RAP2 at MCPH3 (11, 12), CASC5 at MCPH4 (13, 14), ASPM at MCPH5 (15, 16), CENPJ at MCPH6 (17), STIL/SIL at MCPH7 (18), CEP135 at MCPH8(19), and CEP152 at MCPH9 (14, 20) (Table 1). Different mutations have been identified for these loci in Iran and different part of the world. We have ascertained twenty-two consanguineous families with intellectual disability and different ethnic backgrounds from Iran. Ten out of twenty-two families showed primary microcephaly in clinical examination. 

## Materials & Methods

**Clinical examination**

We evaluated 22 families with two or more intellectual disability patients referred to the Genetics Research Center, Tehran, Iran. These families were from various ethnicities and different provinces of Iran. Informed consents were taken from the family members who participated in this study. Ten out of twenty-two families showed primary microcephaly. Consanguinity was observed in 7 out of these 10 families. On examination, head circumferences were -3 to -13 SD below the population age and sexrelated mean values. All parents had normal intelligence scores and normal head circumferences (Table2).

**DNA Extraction and Genotyping**

DNA was extracted from peripheral blood lymphocytes following a standard protocol. A panel of 70 microsatellite markers was selected from the Genome Databases (http://www.gdb.org/ and http:// genome.ucsc.edu/). Population-specific allele frequencies were available for the Iranian population, because of previous studies in Iran. polymerase chain reaction (PCR) amplification of the microsatellite markers are performed(Table3). 

Polyacrylamide gel electrophoresis and standard silver stain protocol were used to visualize the PCR products. When the haplotype at a MCPH locus was found to be homozygous in all affected members of a family, mutation screening was initiated. If different homozygous haplotypes or heterozygous markers were found in the affected individuals, the respective locus would be excluded.

**Sequencing of ASPM**

All 28 exons, and exon/intron splice sites of the ASPM gene (National Center for Biotechnology Information Gen Bank Accession Number AF509326), of the family linked to the MCPH5 locus on chromosome 1q31, were sequenced using a set of 33 PCR primers (designed with the Primer3 software). Sequences were compared with the reference genomic and cDNA sequence (NM_018136).

**Table 1:** A Review of The Previous Studies On Loci For Autosomal Recessive Primary Microcephaly

Locus	Genomic region	Gene	Ethnicity	Reference
MCPH1	8p22-pter	Microcephalin	Northern Pakistani, Iranian	(27, 28)
MCPH2	19q13.1e13.2	WDR62	Northern Pakistani, Indian, Pakistani	(9)
MCPH3	9q34	CDK5RAP2	Northern Pakistani	(29, 30)
MCPH4	15q14	CASC5	Moroccan, Canada	(14)
MCPH5	1q31	ASPM	Northern Pakistani, Turkish, Jordanian Dutch, SaudiArabian, Yemeni, Indian	(15, 31)
MCPH6	13q12.2	CENPJ	Northern Pakistani, Brazilian, Pakistani	(17, 30)
MCPH7	1p32.3ep33	STIL	Indian	(18)
MCPH8	4q12	CEP135	NorthernPakistani	(19)
MCPH9	15q21.1	CEP152	Moroccan, Pakistani	(20, 31)

**Table 2:** Microcephalic Families

**Number**	**Family**	**Linkage results**	**Affected number**	**Additional feature**
1	9000003	Unlinked	2	-
2	9000007	Unlinked	3	-
3	9000013	MCPH5	2	-
4	9000017	Unlinked	3	-
5	9000018	Unlinked	2	-
6	9000039	Unlinked	2	-
7	9000056	Unlinked	3	-
8	9000120	Unlinked	2	-
9	9000140	Unlinked	3	-
10	9000141	Unlinked	2	-

**Table 3:** Standard Procedures Of PCR In A Total Volume Of 30 Ul And Thermal Cycling Conditions

**Cycle step**	**Temp**	**Time**	**Cycle number**
denauration	95°C	5 min	1
Denaturation Annealing Extension	94°C Tm 72°C	40 sec 30 sec 40 sec	30
Final extension	72°C	2 min	1
	4°C	hold	

**Table 4:** Clinical Features of The Microcephalic Family Linked To MCPH5 Locus

**Patient**	**Sex**	**Age** **(yrs)**	**Severity of Intellectual Disability**	**Height (Cm)**	**OFC (Cm)**	**Other features**
Ⅴ :1	Male	14	Severe	143	-13SD(41cm)	-
Ⅴ :2	Male	27	Severe	153	-10SD(45cm)	-

**Fig 1:**
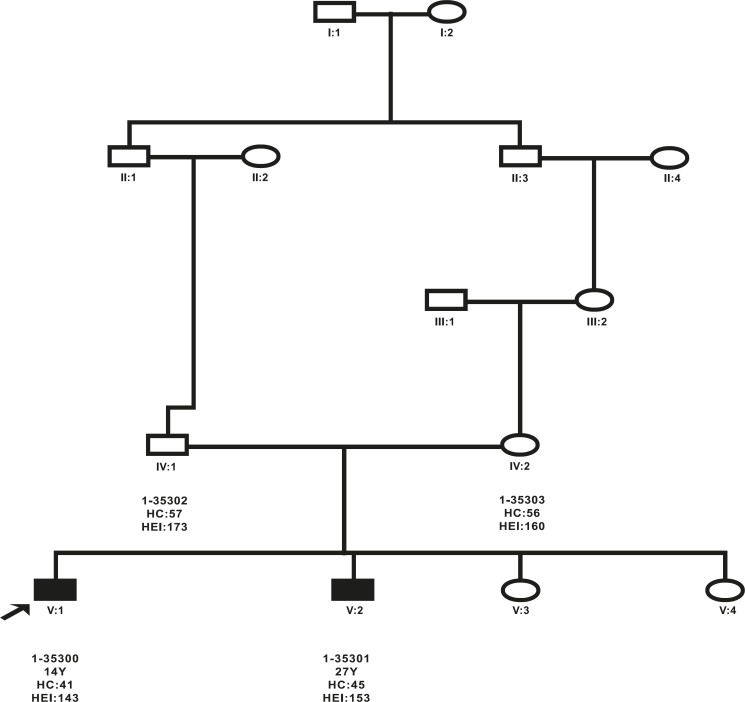
Pedigree of family (9000013) with novel mutations in the ASPM gene; affected males are indicated by filled squares

**Fig 2:**
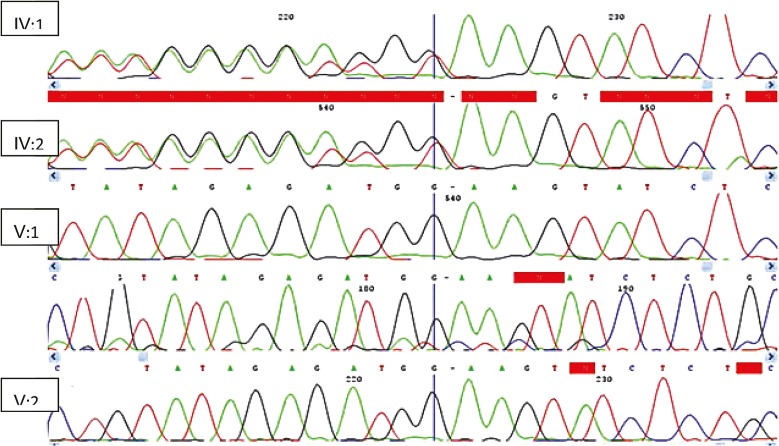
Sequence analysis of the ASPM gene in family (9000013); the upper panel represents the sequence in parents, while the lower panel represents the sequence in the affected individuals

**Fig 3:**
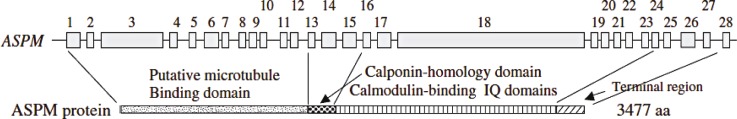
Diagrammatic representation of the exon/intron structure of the ASPM gene according to Bond et al’s study (15)

## Results

**Clinical Findings**

The consanguineous Iranian family with primary MCPH (9000013) had two affected subjects, including two males (Fig.1) with ages varying between 14 and 27 years.

Head circumference of the two affected individuals was 10 to 13 SD below the expected mean for sex and age and they (V: 1 and V:2) had severe intellectual disability (intelligence quotient [IQ] of 30 to 35). They were unable to read or write and did not have basic self-care skills. With the exception of intellectual disability, there were no other neurological and motor development problems. The parents had normal head circumference and normal intelligence (Table4).

**Genotyping and Mutation Analysis**

Linkage was performed using STR markers present within the known MCPH loci. Linkage of a family to MCPH locus was based on the observation that all affected individuals had the same homozygous pattern. One out of ten families showed homozygosity at MCPH5 locus (family [9000013]).

Sequence analysis of exon 8 in 2 affected individuals (V: 1, V: 2) and in parents ( : 1, :2) revealed a deletion of nucleotide (T) in donor site of splicing site (Fig.2). This deletion mutation was present in heterozygous state in the parents. We did not find any linkage for the remaining nine families with primary microcephaly.

## Discussion

The MCPH5 has been shown to be the most prevalent MCPH loci in Iran with a frequency of 13.3% (2). It was also the most common locus among microcephal populations in Pakistan (accounting for 43% to 86% of the loci) (21), and India (33.5% of the loci) (7, 22). 

In this study, one family showed linkage to MCPH5 (ASPM). We detected a novel mutation in the donor splicing site of exon 8 of the ASPM gene. This deletion mutation can alter the ASPM transcript leading to functional impairment of the gene product. 

The ASPM gene with 28 exons is the human ortholog of the Drosophila melanogaster ‘abnormal spindle’ gene (asp), which is essential for normal mitotic spindle function in embryonic neuroblasts (15, 23) (Fig.3). 

ASPM was highly expressed in fetal and adult human tissues with lower levels in adult tissues. The predicted full-length protein contains 3477 amino acids and has a calculated molecular mass of 410 kD. ASPM contains two conserved regions, ASPM N-proximal (ASNP) repeats, and C-terminal calmodulin-binding IQ motifs with variable length. Immuno-staining of cultured human cells revealed that ASPM was localized in the spindle poles during mitosis (24). ASPM gene is essential for symmetric proliferative division of neuroepithelial cells during brain development (16). Postnatally, ASPM expression decreases neurogenesis and upregulation of gliogenesis in the cortex. This expression pattern shows that ASPM is involved in neuron rather than glia production (25). The previously published ASPM mutations comprise deletions of 1–7 base pairs, nonsense mutations, a breakpoint translocation, and intronic splicedonor site mutations (7, 25, 26). 

The remaining nine microcephalic families were not linked to any of the known loci. These families will help to refine the mapping of the other MCPH loci or the genes which have not been identified.

## Notes

**Patient consent: **Obtained.

**Ethics approval: **This study was conducted with the approval of the University of Social Welfare and Rehabilitation Sciences.
